# Osteogenic differentiation of rat bone mesenchymal stem cells cultured on poly (hydroxybutyrate-co-hydroxyvalerate), poly (ε-caprolactone) scaffolds

**DOI:** 10.1007/s10856-021-06615-6

**Published:** 2021-10-30

**Authors:** Ana A. Rodrigues, Nilza A. Batista, Sônia M. Malmonge, Suzan A. Casarin, José Augusto M. Agnelli, Arnaldo R. Santos, William D. Belangero

**Affiliations:** 1 Laboratório de Biomateriais em Ortopedia, Faculdade de Ciências Médicas, Universidade Estadual de Campinas, Campinas, SP Brazil; 2 Centro de Engenharia, Modelagem e Ciências Sociais Aplicadas (CECS), Universidade Federal do ABC, São Bernardo do Campo, SP Brazil; 3 Departamento de Engenharia de Materiais, Universidade Federal de São Carlos, São Carlos, SP Brazil; 4 Centro de Ciências Naturais e Humanas (CCNH), Universidade Federal do ABC, São Bernardo do Campo, SP Brazil

## Abstract

Bioresorbable biomaterials can fill bone defects and act as temporary scaffold to recruit MSCs to stimulate their differentiation. Among the different bioresorbable polymers studied, this work focuses on poly(hydroxybutyrate-co-hydroxyvalerate) (PHBV) and poly(ε-caprolactone) (PCL). Were prepared blends of PHBV and PCL to obtain PHBV based biomaterials with good tenacity, important for bone tissue repair, associated with biocompatible properties of PCL. This study assesses the viability of Vero cells on scaffolds of PHBV, PCL, and their blends and the osteogenic differentiation of mesenchymal stem cells (MSCs). Materials were characterized in surface morphology, DSC and Impact Strength (IS). Vero cells and MSCs were assessed by MTT assay, cytochemical and SEM analysis. MSC osteogenic differentiation was evaluated through alizarin red staining and ALP activity. We found some roughness onto surface materials. DSC showed that the blends presented two distinct melting peaks, characteristic of immiscible blends. IS test confirmed that PHBV-PCL blends is an alternative for increase the tenacity of PHBV. MTT assay showed cells with high metabolic activities on extract toxicity test, but with low activity in the direct contact test. SEM analysis showed spreading cells with irregular and flattened morphology on different substrates. Cytochemical study revealed that MSCs maintained their morphology, although in smaller number for MSCs. The development of nodules of mineralized organic matrix in MSC cultures was identified by alizarin red staining and osteogenic differentiation was confirmed by the quantification of ALP activity. Thus, our scaffolds did not interfere on viability of Vero cells or the osteogenic differentiation of MSCs.

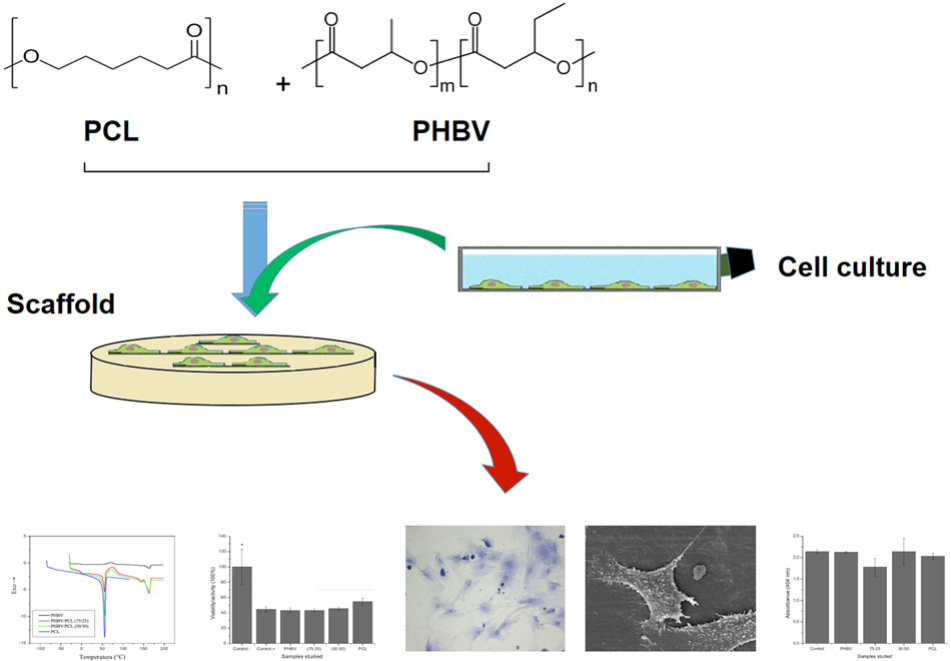

## Introduction

Huge tissue losses due to traumas, infections, tumors, or degenerative diseases have been treated for decades through the removal of the patient’s tissues and substitution of damaged areas by prostheses. Yet, these methods demand revision surgeries, cause physical limitations, and, consequently, decrease the quality of life of such patients [1]. Thus, methods involving engineering to produce tissues with the same properties as the injured ones, leading to more efficient recovery and rehabilitation, are particularly interesting [2, 3].

Regenerative medicine based on tissue engineering is a research field that may positively contribute to that purpose. This investigation line has used mesenchymal stem cells (MSCs) to optimize the regeneration process and reestablish the lost structure and function [4]. The main known sources of MSCs are bone marrow, adipose tissues, peripheral blood, and umbilical cord blood [4, 5]. Studies have shown that, when stimulated by specific signs, MSCs derived from such sources can differentiate into cells of bone and cartilage tissues [4, 6, 7]. The capacity to induce the differentiation of MSCs into other cell types with specific functions allows recruiting compatible cells to the damaged area, modulating and optimizing the tissue regeneration process [4–7].

Studies have sought to elucidate the efficiency of MSCs associated with different biomaterials in the most different clinical applications. Broadly speaking, they aim at clarifying not only the isolation, maintenance, and differentiation mechanisms of MSCs in vitro, but also and mainly their clinical application and interaction with scaffolds, which are possible applications [2–4, 7].

In orthopedics, such biomaterials can fill bone defects and still act as temporary scaffold to recruit MSCs to the damaged place in order to stimulate their differentiation into osteoblasts [8]. Nevertheless, the most appropriate biomaterials to fill these defects and favor the growth of bone cells still need definition.

Among the bioresorbable polymers used for that purpose are polyhydroxyalkanoates (PHAs), which are produced by microorganisms from renewable carbon sources. Among PHAs, we highlight poly(3-hydroxybutyrate) (PHB), poly(hydroxybutyrate-co-hydroxyvalerate) (PHBV), poly(4-hydroxybutyrate) (P4HB), poly(hydroxybutyrate-co-hydroxyhexanoate) (PHBHHx), and poly(3-hydroxyoctanoate) (PHO). Since such compounds can degrade in the presence of body fluids, they are indicated as raw materials to develop orthopedic temporary scaffolds that may substitute the metallic implants conventionally used to fix fractures and substitute bone segments [9]. On the other hand, poly(ε-caprolactone) (PCL) is a bioresorbable material approved by the Food and Drug Administration for use in humans. It has important characteristics, such as biodegradability, moderate undesirable host reactions, compatibility with a wide range of other polymers and good processability. Thus, PCL allows the manufacture of a variety of structures and shapes for tissue engineering [3, 10].

Polymer blends are the physical mixture of two or more polymers, where the properties resulting from the mixture are related to the properties of pure polymers. In this study, we produced blends of PHBV and PCL to obtain biomaterials based PHBV with good tenacity, an important mechanical characteristic for bone tissue substitutes, associated with the plasticizing and biocompatible properties of PCL. The objective was to assess the behavior of such blends in the presence of cell cultures as fibroblasts and MSCs induced by osteogenic differentiation.

## Materials and methods

### Preparation of scaffolds of poly(hydroxybutyrate-co-hydroxyvalerate), poly(caprolactone), and their blends

Were used poly(hydroxybutyrate-co-valerate) (PHBV) containing 12% valerate and polymer PCL type CAPA 6500 - with molar mass (MW) of 50,000 g/mol provided by BIOCYLE (PHB Industrial S/A) (lot FE133) and Solvay, respectively. Specimens of the polymers and their blends were prepared in the Materials Engineering Department of the Federal University of São Carlos. Blends of PHBV/PCL were prepared in 75:25 and 50:50 proportions. The initial mixtures of polymer granules were produced, extruded, and molded into specimens by injection molding. Polymer blends were extruded using a modular co-rotating twin-screw (L/D = 35) Werner & Pfleiderer ZSK 30 extruder and specimens were injected using a fully automated Arburg Allrounder injection molding machine. The different polymer compositions produced were injected to obtain circa 2 mm thick specimens in the form of plates according to method develop by Casarin et al. [10]. These were cut into appropriate sizes, sterilized by (25 KGy) gamma radiation, and submitted to cytotoxicity tests. This study used the following materials: PHBV, PCL, PHBV/PCL 75:25, PHBV/PCL 50:50.

### Vero cell culture

Vero cells, a fibroblastic cell line derived from the kidney of the African green monkey (*Cercopithecus aethiops*), provided by Adolfo Lutz Institute, São Paulo, Brazil, were used. They were cultured in Ham’s-F12 medium (Sigma, St. Louis, MO, USA) supplemented with 10% fetal bovine serum (FBS, provided by Nutricell, Campinas, Brazil), and 1% penicillin/streptomycin (PS, Hyclone) at 37 °C with 5% CO_2_. Vero cell lines are internationally recommended as a standard to study cytotoxicity and cell-substratum interactions with biomaterials [11, 12].

### Isolation and culture of bone marrow mesenchymal stem cells of rats

To obtain MSCs, the femurs, tibias and humeri of Wistar-Kyoto rats were removed. The project was approved by the Ethics Committee on Animal Experimentation of Faculty of Medical Sciences, State University of Campinas (Protocol 1958-1). After their epiphyses were cut off, they were introduced into 5 ml blood collection tubes and centrifuged for 10 min at 400 *g*. The precipitated bone marrow was homogenized using a (2 ml/bone) PBS/EDTA solution, circulated through a (20 µm) filter, placed on 15 ml of Ficoll-Hypaque^®^, and centrifuged for 25 min at 300 g. The fractions of mononucleated cells were collected and centrifuged for 10 min at 200 *g*. After the supernatant was discarded, the precipitate was homogenized in 10 ml of PBS/EDTA. The suspension obtained was washed and centrifuged three times at 200 *g* for 10 min. Cells were then counted in a Neubauer chamber and placed in DMEM medium with low glucose concentration supplemented with 10% FBS and 5% CO_2_.

### Surface morphology of biomaterials specimens by optical microscopy

The morphological analysis of specimens surfaces was performed using a Stereo Microscope (Physis, model SZ40). Photographs were taken from all samples with a digital camera coupled to a stereoscope at ×1.0, ×1.5, ×2.0, ×3.0, and ×4.0 magnification.

### Differential scanning calorimetry (DSC)

DSC analysis was realized by using the equipment TA, DSC Q100, in N2 atmosphere. In the case of PHBV and the blends the temperature was scanned from −30° to 200 °C and for PCL the temperature was changed from −80° to 150 °C, with the heating of 10 °C/min.

### Impact strength

The impact strength (IS) was realized with notched specimens in an equipment Izod Impact Ceast code 6545100, with a 2,0 J pendulum according to ASTM D-256-02 [13]. A set of ten specimens/sample were used to obtain the average value and standard deviation of the IS.

### MTT assay

This assay used the modified Mosmann method [14]. Extracts of the tested materials (PHBV, PCL and their blends) were obtained by incubating them in Ham’s F-12 medium containing 10% FCS at a proportion of 0.2 g/ml medium for 24 h at 5% CO2 and 37 °C. This method is in agreement with ASTM F813-83 for evaluation of biomedical devices [15]. For the extract toxicity assay, Vero cell suspensions (3 × 10^6^ cells/ml) were inoculated into a 96-well cell culture plate (*n* = 5) and incubated at 37 °C for 24 h. After this, the culture medium was replaced by the extract obtained from the tested materials, and the cells were maintained under these conditions for 24 h. For the direct cytotoxicity assay, a suspension of 3 × 10^6^ cells/ml (*n* = 5) was directly cultivated on the materials for 24 h. Ham’s F-12 medium with phenol 0.5% was used as the positive control toxicity and polystyrene extract as the negative control toxicity in both tests. After incubation, the medium was removed and the wells were washed with 200 µl PBS. Next, 200 µl of Ham’s F12 medium with 10 mM of Hepes buffer and 50 µl of thiazolyl blue tetrazolium bromide solution (MTT, Sigma) were added, and the plate was incubated in the dark for 4 h at 37 °C. After that, the medium with MTT was removed, and 200 ll of dimethyl sulphoxide was added. The absorbance curve was determined in a microplate reader (Bio-Rad 550 microplate spectrophotometer) at *k* = 540 nm. Commercial software (MicrocalTM Origin version 6.0) was used for statistical calculation. Student’s *t* test was employed for assessing statistical differences between each sample and controls, while one-way analysis of variance (ANOVA) was employed for assessing statistical differences between all samples. *P* < 0.05 was considered statistically significant.

### Scanning electron microscopy (SEM)

For this assay, 3 × 10^6^ cell/mL of Vero cells and MSCs were inoculated on materials in 100 µL of DEMEM medium supplemented with 10% FBS into a 96-well plate (Corning) containing the three different tested materials and incubated at 37 °C in 5% of CO_2_ for 24 h. After fixation in paraphormaldehyde (Sigma) 2.5% and glutaraldehyde (Sigma) 2.5%, the samples were post-fixed in 1% solution of osmium tetroxide (OsO_4_, Sigma) for 1 h at room temperature in the dark, washed in distilled water, dehydrated in ethanol, critical point dried in CO_2_ (Balzers, CDT 030), coated with gold in a sputter coater (Balzers CTD 050), viewed and photographed on an electron microscope (JEOL 5800).

### Cytochemical analysis

Vero and MSCs cells were cultivated in contact with PCL, PCL/PHBV 50:50, PCL/PHBV 75:25 and PHBV. To do so, 3 × 10^6^ cells/ml (*n* = 4) suspended in low glucose DMEM medium with 10% FBS were inoculated on the materials and the plate was cultivated for 24 h at 37 °C. The cells were then fixed in formaldehyde 10% for 24 h and stained with toluidine blue (TB) at pH 4.0 to detect the presence of nucleic acids and glycosaminoglycan acids [16, 17]. The images were obtained with a Leica inverted microscope (model DM IL, Leica Microsystems, Wetzlar, Germany) with 10× and 20× objective lens.

### Induction of osteogenic differentiation

MSCs were inoculated in 24-well plates and osteogenic differentiation was induced according to Neuhuber et al. [18]. After 48 h, material samples were added in the wells and the culture medium was replaced with osteogenic induction medium consisting of low-glucose DMEM with 15% FBS, 1% penicillin/streptomycin, 100 nM dexamethasone, 50 μM ascorbate-2-phosphate, and 10 mM glycerol-phosphate. As a control, MSCs were cultured without any material. The medium was changed every 3 days during 21 days. After this period, the cells were tested for alkaline phosphatase activity and development of mineralized bone matrix.

### Alkaline phosphatase assay

Alkaline phosphatase enzyme activity (ALP) was assessed with a specific kit (Sigma Fast p-nitrophenyl phosphatase Tablets N1891) according to the manufacturer’s recommendations. After 21 days of osteogenic differentiation, 50 μl of the differentiation medium contained in the 24-well plate were transferred into a 96-well plate and incubated for 2 h at 37 °C. Next, 200 μl of the ALP substrate solution were added and the plate was maintained in the dark for 30 min at room temperature. Absorbance was read at 405 nm. Statistical significance (*P* < 0.05) was assessed using one-way analysis of variance (ANOVA).

### Alizarin red staining

After 21 days of osteogenic differentiation, the cells were fixed in paraformaldehyde 4% for 10 min, washed in distilled water, stained with Alizarin Red S (ARS) for 2 min, washed again in distilled water, differentiated in ethanol 95% and hydrochloric acid 100% for 15 s and placed in distilled water. Pictures were shot with an inverted Leica microscope (model DM IL) with 10× and 20× objective lens. ARS activity was quantified using the colorimetric method described by Gregory et al [19]. Water was drained and the plate was kept at room temperature until complete dryness was achieved. Next, 280 μl of acetic acid 10% were added and the plate was shaken for 30 min. Due to the resolution of our equipment, the contents of each experimental sample were pooled and then transferred to Eppendorf tubes, which were heated to 85 °C for 10 min and then cooled down in ice for 5 min. Therefore, the assay performed were semiquantitative. After the tubes were centrifuged at 16.000 *g* for 20 min, 100 μl of the supernatant were placed in a tube with 40 μl of ammonium hydroxide 10%. The final solution was transferred into a 96-well plate. Absorbance was read at 405 nm.

## Results

### Surface morphology of studied biomaterials

**Fig. 1:**
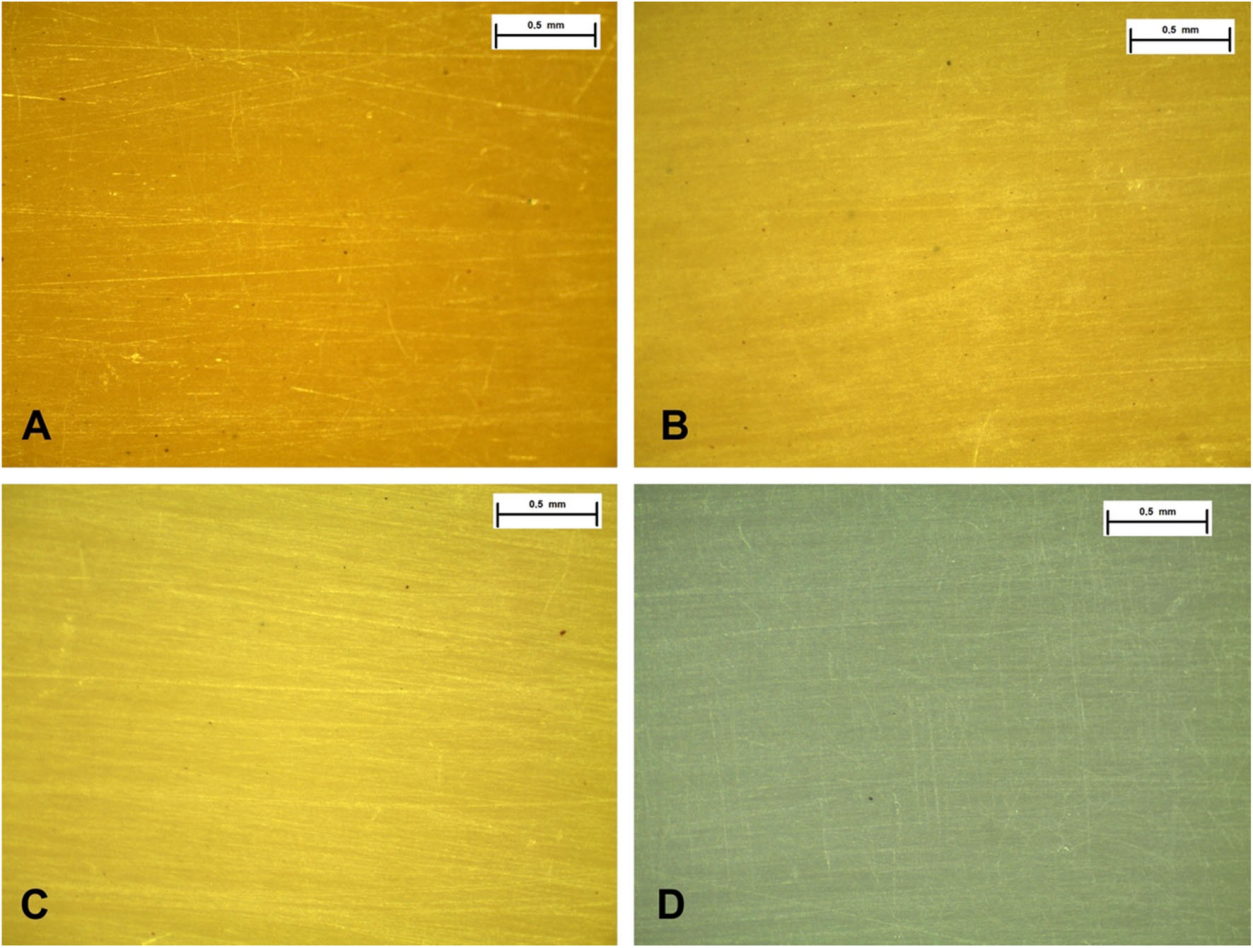
Optical microscopy images of (**A**) PHBV, (**B**) PHBV/PCL 75:25, (**C**) PHBV/PCL 50:50 and (**D**) PCL. Note the scratchs characteristics of the injection process, which resulted in some roughness. The material surface is flat, resembling the culture plate’s surface. Scale bar = 0.5 mm

Figure 1 shows the images of the specimen’s surface that allowed verifying the presence of risks characteristics of the injection process, which resulted in some roughness onto surface materials. In general, the surface is flat, resembling the surface of the culture plates.

### Thermal behavior of studied biomaterials by DSC

**Fig. 2:**
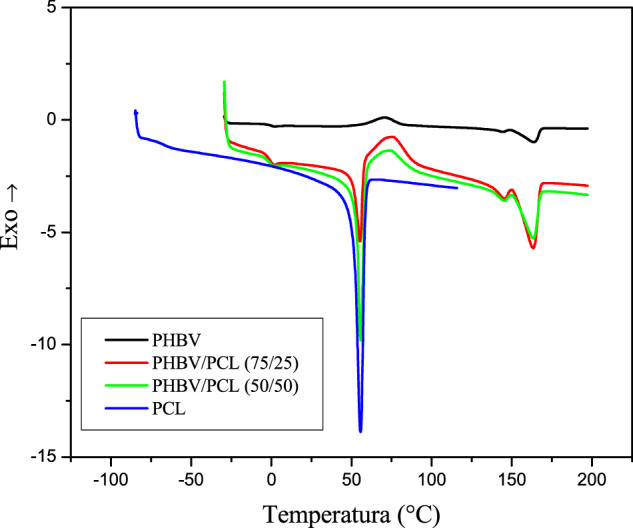
DSC curves for 2° heating: behavior compared between PHBV, PCL and PHBV/PCL blends

The Table 1 shows the values of melting temperature (Tm) and transition glass temperature (Tg), beyond of melting enthalpy and crystallinity of the studied samples. The crystallinity percentage of PHBV and PCL were calculated by using the data of theoretical value of fusion enthalpy of PHB hypothetically 100% crystalline (ΔHf_100%_ = 146 J/g) [20] and of PCL hypothetically 100% crystalline (ΔHf_100%_ = 136 J/g) [21], as well as the actual values of pure PHBV and PCL. The Fig. 2 illustrates the DSC curve of the studied materials. Considering the obtained data is possible to note that the blends presented two distinct melting peaks, one near around 64 °C indicating the PHBV phase and the other near around 55 °C, characteristic of the do PCL.

**Table 1:** Values of T_m_, T_g_, ΔH_m_ and crystallinity

Polymers	PHBV				PCL		
	T_m_ (°C)	ΔH_m_ (J/g)	%C^a^	T_g_ (°C)	T_m_(°C)	ΔH_m_ (J/g)	%C^a^
PHBV	164.1	46.8	32.4	−0.2	–	–	–
PHBV/PCL (75/25)	164.1	37.1	33.9	−0.6	56.2	18.2	54.7
PHBV/PCL (50/50)	163.6	20.3	28.2	−1.5	55.3	35.7	52.8
PCL	–	–	–	–	55.3	68.3	51.1

^a^Percentage of crystallinity of the pure polymers or polymers in the blend (standardized by the experimental levels of the PHBV and PCL in the blends)

### Impact strength of studied biomaterials

The Table 2 describes the values obtained in the impact resistance test. According to these results, it was observed that the resistance value of the impact of the pure PHBV is lower than the value showed by the pure PCL. For blends studied was noted that the blend PHBV/PCL (75:25) showed a value of impact resistance higher than the value presented by the pure PHBV, with an increase of approximately 30% while the blend PHBV/PCL (50:50) showed a value similar to the arithmetic mean of the values presented by the PHBV and PCL.

**Table 2:** Results of Izod impact resistance, notched

Polymers	Average (J/m)	Standard deviation (%)
PHBV	28.6	2.0
PHBV/PCL (75/25)	37.4	2.8
PHBV/PCL (50/50)	119.0	3.1
PCL	202.4	4.9

### MTT assay

**Fig. 3:**
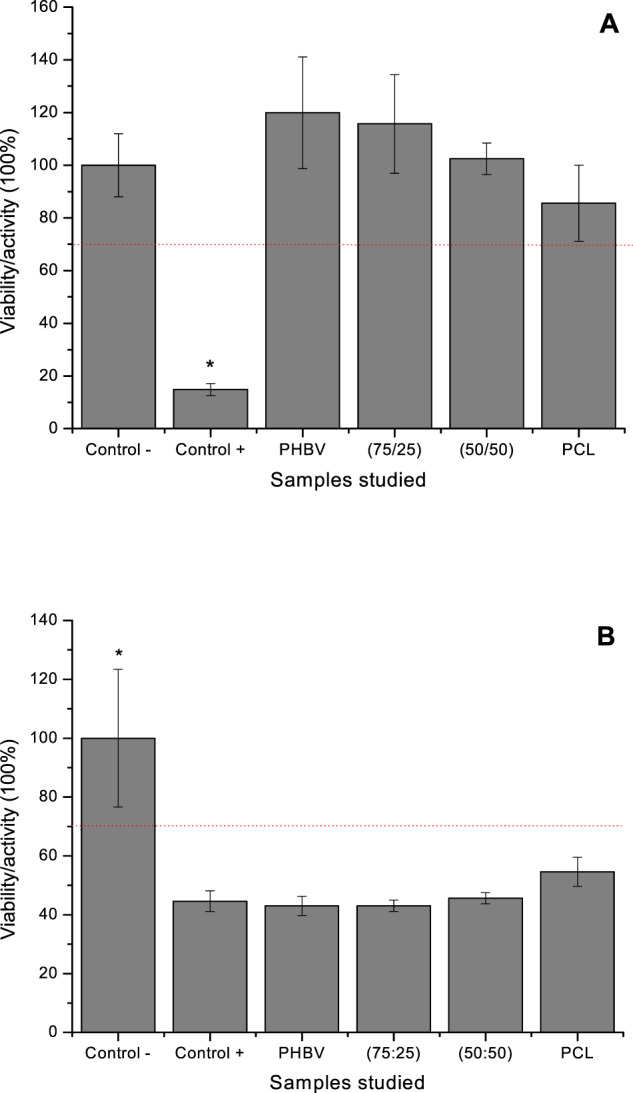
MTT assay of PHBV, PCL and their blends with Vero cells. **A** cytotoxicity by extract and (**B**) direct cytotoxicity. Negative control = polystyrene extract; positive control = phenol 0.5%. The * indicate statistically significant differences (*p* > 0.05) between the samples and the negative control of toxicity

The MTT assay showed that in contact with polymer extracts, the number of adherent viable cell was similar to negative control (Fig. 3A). According to the Student’s *t* test, there are no statistically significant differences between any sample and negative control (no toxic) (*p* < 0.05), except positive control. On the other hand, in direct contact, the quantity of viable cells on different polymers was similar to positive control (Fig. 3B).

### Scanning electron microscopy (SEM)

**Fig. 4:**
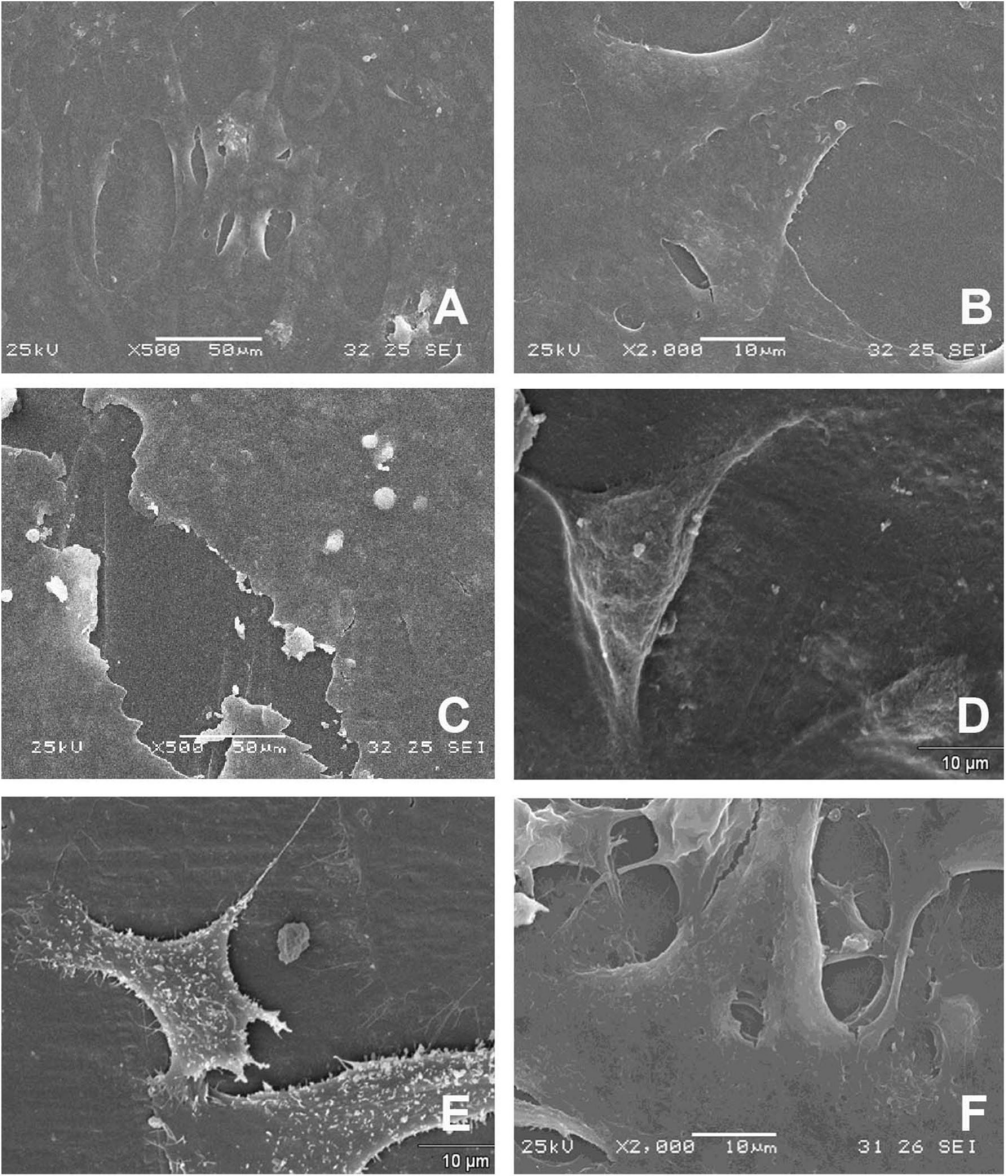
Scanning electronic microscopy (SEM) of Vero Cells cultured by 24hs on experimental conditions: **A** and **B** Control on glass coverslip; **C** PHBV; **D** PHBV/PCL (75:25); **E** PHBV/PCL (50:50) and **F** PCL. Scale bar = 10 or 50 µm

For Vero cells analysis, we could se on glass coverslip flattened and elongated cells forming a semi confluent layer (Fig. 4A e 4B). On PCL we found cells flattened with irregular morphology (Fig. 4C). On PCL/PHBV (50:50) blends we could see spreading and elongated cell, some times with some thin cell extensions, on substrates. Vesicles and microvillies were also found in some cells (Fig. 4D). The cells cultured on PCL/PHBV (75:25) blends showed irregular morphology, flattened and retracted with cell prolongations on materials (Fig. 4E). On PHBV, cells with a very flattened morphological pattern were found (Fig. 4F). For Vero cells, we did not found fibrilar material around cells in any sample studied.

**Fig. 5:**
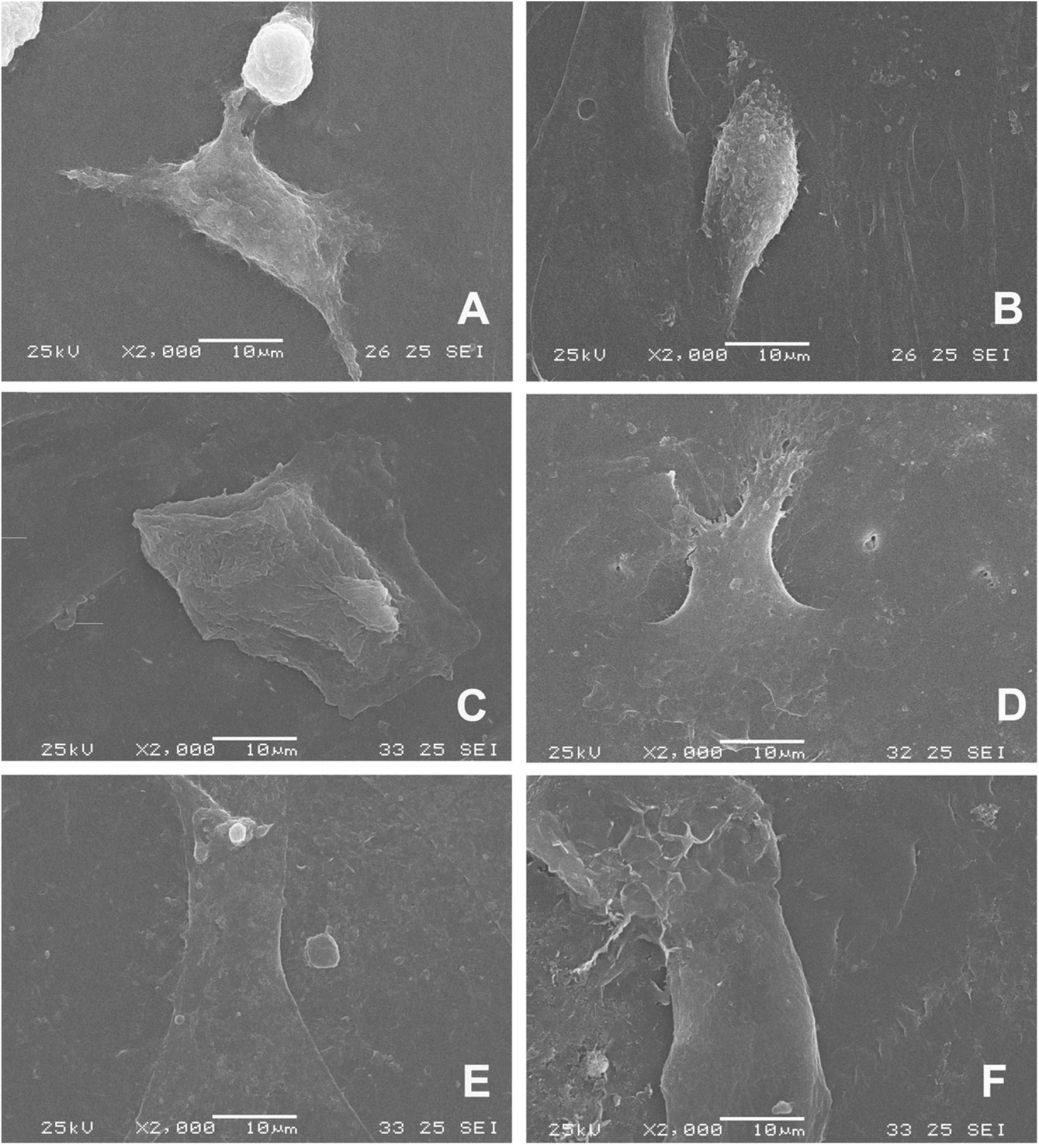
Scanning electronic microscopy (SEM) of MSCs cultured by 24hs on experimental conditions: **A** and **B** Control on glass coverslip; **C** PCL; **D** PHBV/PCL (50:50); **E** PHBV/PCL (75:25) and **F** PHBV

It was noticed by MTT assay a small amount of MSCs adhered on the surface of the studied materials. With the use of SEM, few cells were found with a great spaced around them (data not shown). Thus, the SEM objective was to evaluate the cell morphology on polymers. On the glass coverlisp, it was noticed the presence of spread cells with varied morphology and star-shaped, oblong and almost bipolar aspect (Figs. 5A e 2B). On PCL, cells with irregular morphology were noticed, mainly with elongated cells (Fig. 2C). On PHBV/PCL (50:50) we found much-flattened cells (Fig. 5D). In PHBV/PCL (75:25) we could observed some star-shaped spread cells on material surface (Fig. 5E). Finally, on the PHBV surface, flattened cells were noticed. No cellular extensions could be observed (Fig. 5F).

### Cytochemical analysis

**Fig. 6:**
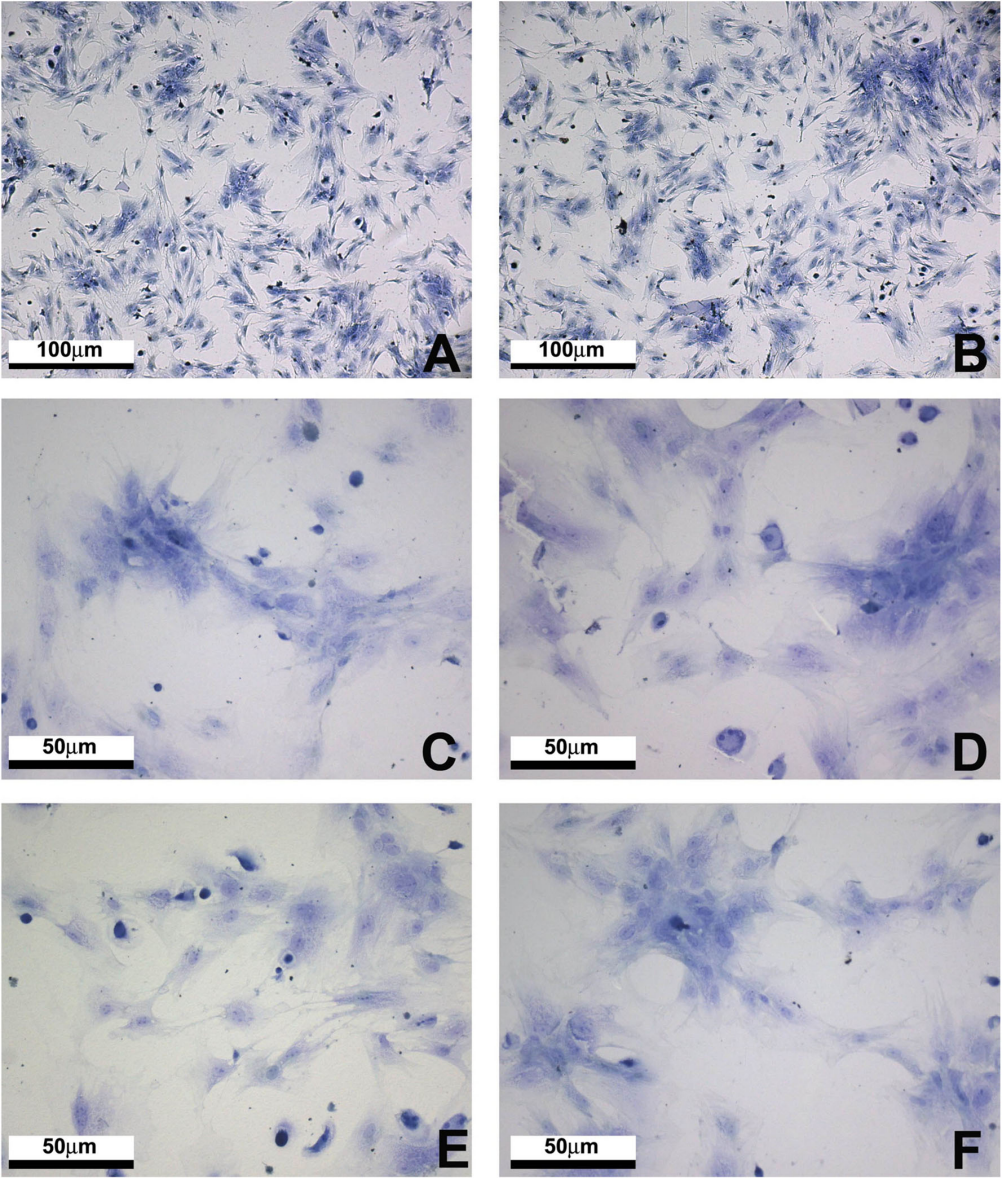
Cytochemical analysis of MSCs cultured cultured by 24hs in different experimental conditions: **A** and **B** Control on glass coverslip; **C** PCL; **D** PHBV/PCL (50:50); **E** PHBV/PCL (75:25) and **F** PHBV. Toluidine Blue (TB) stain. Scale bar = 100 µm or 50 µm

For Vero cells, TB staining showed confluent spread and many cells adhered to the plate. They showed the same growth pattern as negative control. Regardless of the type of biomaterial, strongly basophilic cells with slightly metachromatic nuclei and evident nucleoli, which characterize cellular activity, were detected. For MSCs, after 24 h culture, TB staining showed typical behavior of these cells. At the semi-confluence stage, oblong, occasionally elongated or star-shaped morphology was noticed and, in agglomeration regions, rather contracted cells with a polygonal aspect were seen. The same pattern of cytoplasmic basophilia with a slight nucleolus metachromasia was observed. No influence of the biomaterial types on the activity of the cells in contact with them was noticed. No variations on cell cytochemical results induced by biomaterials were found (Figs. 6A–3F).

### Alkaline phosphatase

**Fig. 7:**
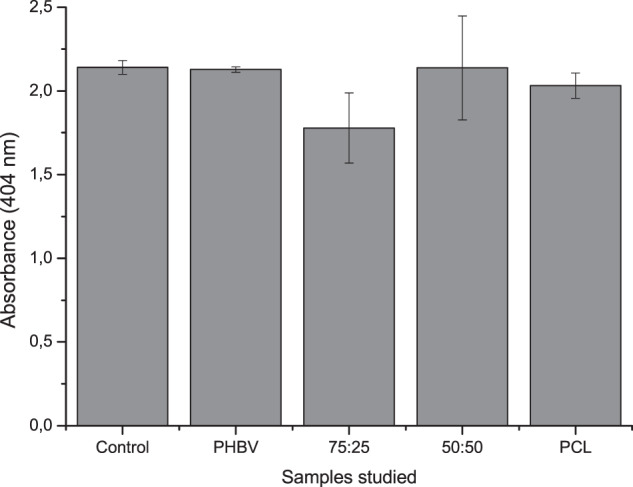
Quantification of alkaline phosphatase activity (ALP) of MSC cells after 21 days of osteogenic differentiation. Control = same culture conditions without scaffolds. There is no statistically significant differences (*p* > 0.05) on the samples

Figure 7 shows the semi-quantitative analysis of ALP enzyme activity, a commonly used marker for bone differentiation. In all the different experimental conditions investigated, the production of this enzyme was observed. We did not find significant differences among samples. Thus, the different polymers did not interfere (stimulating or repressing) the production of ALP.

### Alizarin red staining

**Fig. 8:**
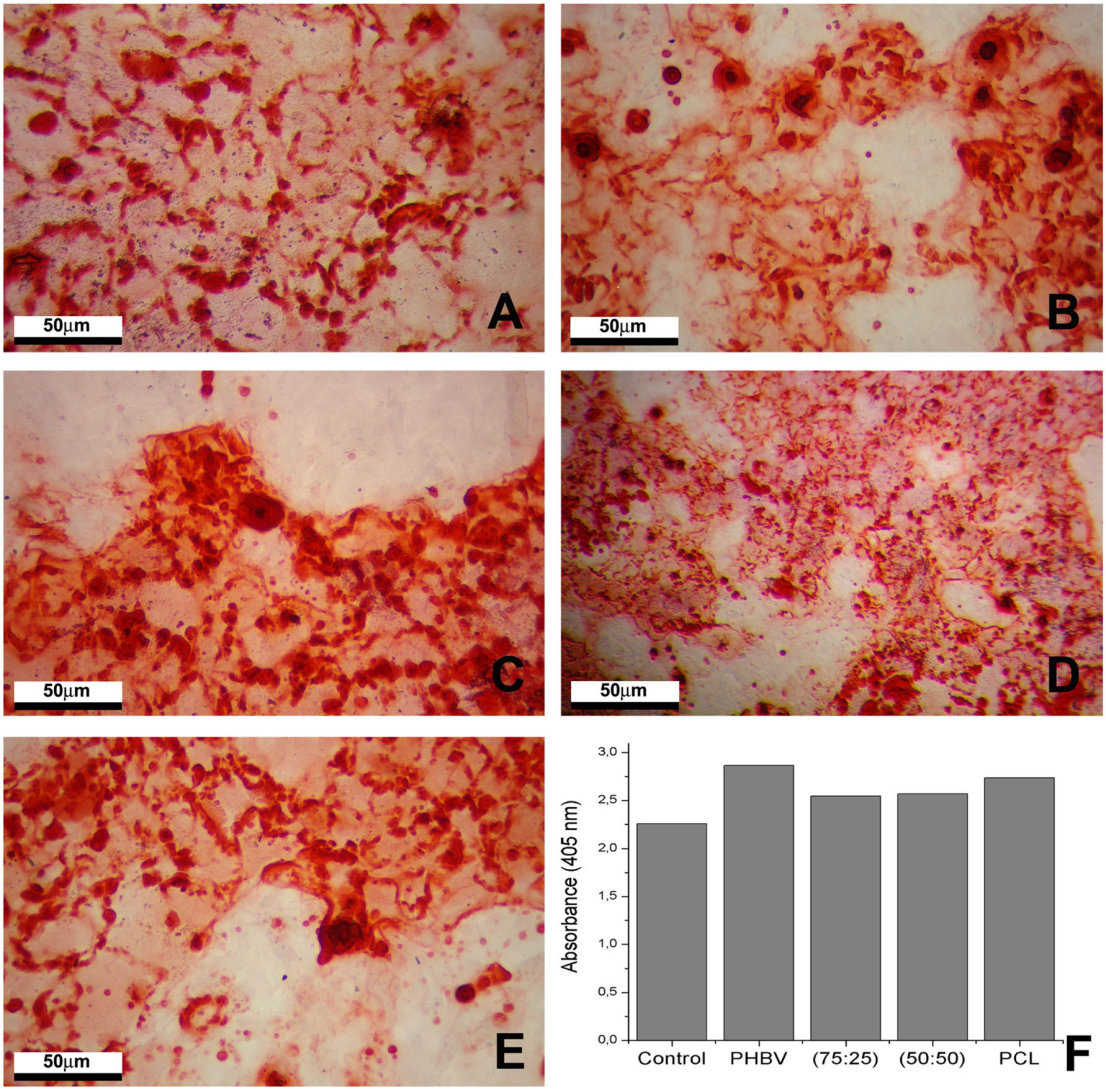
Images of the MSC cells after 21 days of osteogenic differentiation stained with alizarin red (ARS). **A** Control; **B** PHBV; **C** PHBV/PCL (75:25) and **D** PHBV/PCL (50:50); **E** PCL; **F** Quantification of mineralized matrix of MSC cells by colorimetric method of extraction of alizarin red staining (ARS). Control = same culture conditions without scaffolds. Scale bar = 50 µm

The incorporation of ARS after 21 days of osteogenic differentiation indicated the development of mineralized bone matrix. Figure 8 shows the development of mineralization nodules submitted to osteogenic differentiation under contact culture conditions on all the polymers used as substrates. The quantification of the produced matrix is presented in Fig. 8F, where one can see that, in all the studied conditions, the amount of produced material was similar to that of the control. We did not find differences among samples.

## Discussion

Cultured bone marrow MSCs hold a potential to transform into bone and cartilage cells. This transformation occurs in well-defined conditions [2–7]. When cultured in the presence of some biomaterials these cells show capacity to form bone tissue and even ectopic bone formation [22–25]. Thus, the search for new materials with such properties in the presence of MSCs has a significant clinical importance.

For material characterization, however studied materials had no pore structure we could observe an irregular surface of samples for maintain cell culture. The DSC curves showed of two distinct melting peaks indicating the immiscibility of the PHBV/PCL blends. It was also verified a small decrease of the Tm as result of the increase in the amount of PCL in the blend. Otherwise, with respect to the crystallinity of the material, was observed that a significant reduction occurs only for high percentages of PCL in the blend.

According to IS test, it was observed that the resistance value of the impact of the pure PHBV is lower than the value showed by the pure PCL. Considering that the Tg of PCL is ~73 °C below the ambient temperature, its amorphous phase is totally rubbery, making it a very flexible material with high impact resistance. For blends studied was noted that the blend PHBV/PCL presented intermediate values of IS, between the pure polymers, confirming that the mixture of PCL with PHBV is an alternative to make feasible the use of these biomaterial in orthopedics. Taken together, the characterization data indicates that substrates were shown to be stable for the culture conditions planned in those assays. Because of its biocompatibility with human tissues and because it is of natural origin, PHBV is shown with a promising polymer for tissue engineering and a viable alternative in the repair of bone lesions [26, 27]. Although PHBV is considered a polymer with high potential for biomedical applications, its high crystallinity restricts these applications. The use of PHBV blends with PCL is a proposal to increase the tenacity of PHBV and the biological evaluation of this compound may be important and of great relevance in the context of tissue engineering applied to the treatment of osteochondral defects.

Regarding cell viability, the results of the MTT assay demonstrated there are no toxicity by extracts. On the other hand, on direct toxicity assay, we found a similar cell behavior to toxic control. On the other hand, it was observed spreading cells (Vero and MSCs) on SEM analysis forming a monolayer on polymers in the 24 h. We also found a MSCs monolayer on cytochemistry and differentiation assays. This indicates that the materials are non-toxic. If there were toxicity, cell growth would not be observed. Thus, the data were interpreted as an initial difficulty of interaction between cells and polymers. The MTT assay does not indicate toxicity per se. It is an indicator of cellular mitochondrial activity [14]. For this reason, it is commonly and used as an indirect measure of cytotoxicity, but also for cell adhesion and proliferation. Therefore, the MTT trial needs to be analyzed in a broad context. In anchorage-dependent cells, this difficulty may be reflected in cellular activity measured by MTT and, therefore, a low initial interaction with substrata.

Cell adhesion and spreading represent the beginning of the interaction between biomaterial and cell [28]. In this study, the morphology of Vero cells cultured in contact with PHBV, PCL, PHBV/PCL 75:25, and PHBV/PCL 50:50 and assessed by SEM and cytochemistry showed they maintained their morphology, allowing normal cellular adhesion and spreading. In addition, bone marrow MSCs presented typical morphological patterns with scattered, stellate cells although in lesser number when compared to Vero cells. This result was expected, to a certain extent, since MSCs slower adhesion and division kinetics than Vero cells. Thus, despite the slow initial interaction detected by the MTT, once adhered, the cells were able to spread over the studied polymers, presenting a morphological pattern compatible with that expected for anchorage-dependent cells.

For cytochemistry, TB (basic stain that forms electrostatic links with the acid radicals present in the tissues) revealed cells with marked basophilia, slightly metachromatic nuclei, and evident nucleoli, indicating the presence of cell activity. Such activity has been identified in the DNA, RNA, rRNA, and in glycosaminoglycans, since TB combines with the PO_4_^−^, SO_4_^−^, COO^−^ groups, which are present in these structures at pH 4.0 [14, 15]. These results agree with previous publications reporting that Vero cells on different bioresorbable polymers, among them the PHBV [29]. More recently, extensive cytochemical evaluation has been conducted with fibroblastic cells grown in PHBV/PCL blends. The results presented herein are consistent with those previously described [30].

Besides MSCs identification based on morphological and phenotypic characteristics, the ability to form expanded cultures of different cell types is one of the criteria available for identifying and distinguishing MSCs cells already committed to a particular path of differentiation. After 21 days inducing the osteogenic differentiation, ALP concentration was dosed and the formation of inorganic nodules was revealed by the impregnation of ARS, which fixes wherever mineralization takes place. ALP is used as an indicator of osteogenic differentiation, while ARS staining identifies the product of this osteogenic differentiation, a mineralized matrix, and quantifies the deposit of these inorganic nodules through colorimetric extraction [19, 31–34]. Our results indicate ALP activity and the presence of mineralized nodules, thus confirming osteogenic differentiation in presence of PCL/PHBV 50:50, PCL/PHBV 75:25, PCL, and PHBV. Such findings agree with previous reports indicate that biodegradable materials, such as poly lactic acid (PLA), poly glycolic acid, PCL and PHBV are being developed as scaffolds to induce bone formation [9, 26, 32, 35, 36].

## Conclusions

In conclusion, the polymers showed characteristics of immiscible blends and the addition of PCL is an alternative to increase PHBV tenacity. The PCL/PHBV 50:50, PCL/PHBV 75:25, PCL and PHBV not only did not interfere in the normal biology of the cells tested. We did not observe changes in the cytochemical and growth pattern of Vero cells or MSCs, although the initial adhesion was slower in the tested polymers. The studied materials also did not interfere negatively in the differentiation pattern of MSCs, allowed them to behave as bone cells, producing ALP and mineralized matrix. Such findings open new use perspectives for these materials in the treatment of bone defects or flaws in humans.

## References

[1] Santos AR Jr, Zavaglia CAC. Tissue Engineering Concepts. In: Hashmi S (ed.) Reference Module in Materials Science and Materials Engineering. Oxford: Elsevier; 2016. 10.1016/B978-0-12-803581-8.04141-2.

[2] Madl CM, Heilshorn SC, Blau HM (2018). Bioengineering strategies to accelerate stem cell therapeutics. Nature..

[3] Kim HD, Amirthalingam S, Kim SL, Lee SS, Rangasamy J, Hwang NS (2017). Biomimetic materials and fabrication approaches for bone tissue engineering. Adv Healthc Mater..

[4] Lin H, Sohn J, Shen H, Langhans MT, Tuan RS (2019). Bone marrow mesenchymal stem cells: aging and tissue engineering applications to enhance bone healing. Biomaterials..

[5] Zheng Y, Xiong W, Su K, Kuang S, Zhang Z (2013). Multilineage differentiation of human bone marrow mesenchymal stem cells in vitro and in vivo. Exp Ther Med.

[6] Ullah I, Subbarao RB, Rho GJ (2015). Human mesenchymal stem cells - current trends and future prospective. Biosci Rep..

[7] Guillot P, Cui W, Fisk NM (2007). Stem cell differentiation and expansion for clinical applications and tissue engineering. J Cell Mol Med.

[8] Henkel J, Woodruff MA, Epari DR, Steck R, Glatt V, Dickinson IC (2013). Bone regeneration based on tissue engineering conceptions - A 21st century perspective. Bone Res.

[9] Elmowafy E, Abdal-Hay A, Skouras A, Tiboni M, Casettari L, Guarino V (2019). Polyhydroxyalkanoate (PHA): applications in drug delivery and tissue engineering. Expert Rev Med Devices..

[10] Casarin SA, Malmonge SM, Kobayashi M, Agnelli JAM (2011). Study on in vitro degradation of bioabsorbable polymers poly(hidroxybutyrate-co-valente) (PHBV) and Poly(caprolactone) (PCL). J Biomater Nanobiotech..

[11] ISO 10993-5. 2009. Biological evaluation of medical devices. Part 5: Tests for cytotoxicity: in vitro methods, 3th ed.

[12] Kirkpatrick CJ (1992). Biological testing of materials and medical devices - A criticalview of current and proposed methodologies for biocompatibility testing: cytotoxicity in vitro. Reg Aff.

[13] ASTM D256-10(2018), Standard Test Methods for Determining the Izod Pendulum Impact Resistance of Plastics, ASTM International, West Conshohocken, PA, 2018.

[14] Mossmam TJ (1983). A rapid colorimetric assay of cellular growth and survival: application to proliferation and cytotoxicity assays. J Immunol Methods..

[15] ASTM F813-83(1996)e1, Standard Practice for Direct Contact Cell Culture Evaluation of Materials for Medical Devices, ASTM International, West Conshohocken, PA, 2001.

[16] Lison L (1960). Histochemie et Cytochemie Animales - Principles et Methodes.

[17] Vidal BC, Mello MLS (2019). Toluidine blue staining for cell and tissue biology applications. Acta Histochem..

[18] Neuhuber B, Swanger SA, Howard L, Mackay A, Fischer I (2008). Effects of plating density and culture time on bone marrow stromal cell characteristics. Exp Hematol.

[19] Gregory CA, Gunn WG, Peister A, Prockop DJ (2004). An Alizarin red-based assay of mineralization by adherent cells in culture: comparison with cetylpyridinium chloride extraction. Anal Biochem..

[20] Barhan PJ, Keller A (1985). A relationship between microstructure and mode of fracture in polyhydroxybutyrate. J Pol Sci – Part A: Pol Chem.

[21] Avella M, Errico ME, Rimedio R, Sadocco P (2002). Preparation of biodegradable polyesters/high-amylose-starch composites by reactive blending and their characterization. J Appl Pol Sci.

[22] Oryan A, Kamali A, Moshiri A, Baghaban Eslaminejad M (2017). Role of mesenchymal stem cells in bone regenerative medicine: what is the evidence?. Cells Tissues Organs.

[23] Yousefi A-M, James PF, Akbarzadeh R, Subramanian A, Flavin C, Oudadesse H (2016). Prospect of stem cells in bone tissue engineering: a review. Stem Cells Inter.

[24] Zhang Y, Xing Y, Jia L, Ji Y, Zhao B, Wen Y (2018). An in vitro comparative study of multi-sources derived mesenchymal stem cells for bone tissue engineering. Stem Cells Dev.

[25] Fennema EM, Tchang LAH, Yuan H, van Blitterswijk CA, Martin I, Scherberich A (2017). Ectopic bone formation by aggregated mesenchymal stem cells from bone marrow and adipose tissue: a comparative study. J Tis Eng Reg Med..

[26] Ke Y, Wang YJ, Ren L, Zhao QC, Huang W (2010). Modified PHBV scaffolds by in situ UV polymerization: Structural characteristic, mechanical properties and bone mesenchymal stem cell compatibility. Acta Biomaterialia.

[27] Dias M, Antunes MCM, Santos AR, Felisberti MI (2008). Blends of poly(3-hydroxybutyrate) and poly(p-dioxanone): miscibility, thermal stability and biocompatibility. J Mater Sci Mater Med..

[28] Palacio MLB, Bhushan B (2012). Bioadhesion: a review of concepts and applications. Philos Trans R Soc A..

[29] Santos AR, Ferreira BMP, Duek EAR, Dolder H, Wada MLF (2005). Use of blend of bioabsorbable poly (L-lactic acid)/poly (hydroxybutyrate-co-hydroxyvalerate) as surface for Vero cell cultured. Braz J Med Biol Res..

[30] Baptista-Perianes A, Malmonge SM, Simbara MMO, Santos AR (2019). In vitro evaluation of PHBV/PCL blends for bone tissue engineering. Mater Res..

[31] Bruder SP, Jaiswal N, Haynesworth SE (1997). Growth kinetics, self-renewal, and the osteogenic potential of purified human mesenchymal stem cells during extensive subcultivation and following cryopreservation. J Cell Biochem..

[32] Di Liddo R, Paganin P, Lora S, Dalzoppo D, Giraudo C, Miotto D (2014). Poly-ɛ-caprolactone composite scaffolds for bone repair. Int J Mol Med.

[33] Rodrigues AA, Batista NA, Bavaresco VP, Baranauskas V, Ceragioli HJ, Peterlevitz AC (2012). Polyvinyl alcohol associated with carbon nanotube scaffolds for osteogenic differentiation of rat bone mesenchymal stem cells. Carbon..

[34] Blair HC, Larrouture QC, Li Y, Lin H, Beer-Stoltz D, Liu L (2017). Osteoblast differentiation and bone matrix formation in vivo and in vitro. Tis Eng Part B..

[35] Ghassemi T, Shahroodi A, Ebrahimzadeh MH, Mousavian A, Movaffagh J, Moradi A (2018). Current concepts in scaffolding for bone tissue engineering. Arch Bone Jt Surg..

[36] Gao C, Peng S, Feng P, Shuai C (2017). Bone biomaterials and interactions with stem cells. Bone Res.

